# A Diffusion-Based Time-Frequency Dual-Stream Contrastive Learning Model for Multivariate Time Series Anomaly Detection

**DOI:** 10.3390/e28040448

**Published:** 2026-04-15

**Authors:** Kuo Wu, Changming Xu, Ranran Zhang, Wei Lu, Yuan Ma, Ende Zhang, Kaiwen Tan

**Affiliations:** 1 School of Computer and Communication Engineering, Northeastern University, Qinhuangdao 066004, China; wuk@mails.neu.edu.cn (K.W.); zhangrr@mails.neu.edu.cn (R.Z.); luwei@mails.neu.edu.cn (W.L.); tankaiwen@mails.neu.edu.cn (K.T.); 2 China Academy of Railway Sciences Corporation Limited, Beijing 100081, China; 3 School of Computer Science and Engineering, Northeastern University, Shenyang 110819, China

**Keywords:** time series, anomaly detection, diffusion models, unsupervised learning, temporal-frequency analysis

## Abstract

Multivariate time series anomaly detection holds critical application value in key domains such as industrial system monitoring, financial risk management, and medical surveillance. However, existing approaches face two major challenges: reconstruction-based or prediction-based models tend to adapt to anomalous patterns during training, thereby weakening the distinction between normal and abnormal samples; furthermore, the non-stationary nature of time series leads to distribution shifts between training and testing data, impairing model generalization. To address these issues, this paper proposes the TFCID model. The model innovatively leverages diffusion principles to effectively impute missing time series data while capturing significant frequency-domain features. In the temporal processing stream, an unconditional diffusion model combined with imputation masking is employed to achieve high-precision imputation of randomly missing values, effectively preventing anomalies from interfering with model training. In the frequency-domain processing stream, an amplitude-aware frequency-domain masked autoencoder is introduced to specifically capture periodic or trend-based pattern anomalies. The model mitigates distribution shift by constraining the discrepancy between temporal and frequency-domain representations via adversarial contrastive learning, and uses this discrepancy as a robust anomaly scoring metric. Experimental results on five public benchmark datasets show that TFCID significantly outperforms state-of-the-art methods in detection accuracy (F1-Score), validating its effectiveness in anomaly detection tasks.

## 1. Introduction

With the rapid development of Internet of Things (IoT) and cloud computing technologies, various sensors and systems continuously generate massive amounts of multivariate time series data. These data serve as the core basis for monitoring system health status, detecting fraudulent activities, and providing early warnings for potential failures [1,2]. Against this backdrop, time series anomaly detection has attracted increasing attention. The objective of this task is to identify data points or subsequences that deviate significantly from normal behavioral patterns within complex temporal data, which is of practical importance for predictive maintenance and reliable system operation [3,4].

Despite the pressing application demands, constructing accurate and stable anomaly detection models remains a significant challenge. First, in real-world scenarios, anomaly events are often low in frequency and diverse in patterns, making it difficult to collect sufficient and reliable labeled samples. Consequently, unsupervised learning approaches have gradually become the mainstream choice for time series anomaly detection [5]. Second, multivariate time series contain complex spatio-temporal dependencies, encompassing both temporal autocorrelation within variables and cross-correlation among variables, which imposes high demands on the model’s representation capabilities [6]. Existing unsupervised methods can be broadly categorized into three types: reconstruction-based [7,8,9], prediction-based [3,10,11], and the more recent imputation-based approaches [12,13,14]. Reconstruction-based methods aim to learn an encoder-decoder model that maps input data to a lower-dimensional space and then reconstructs it, operating under the assumption that normal data yield small reconstruction errors, whereas anomalies produce large ones. Prediction-based methods leverage historical information to forecast future values and utilize the prediction error as an anomaly indicator. Imputation-based approaches fill randomly missing parts of the sequence and detect anomalies using the imputation error. However, these three paradigms all exhibit inherent limitations: reconstruction models may “perfectly” reconstruct anomalies due to powerful decoder capabilities, thereby weakening the anomaly signal [15,16]; prediction models can suffer from unstable prediction errors when faced with highly uncertain future values, compromising detection robustness [17]; and traditional imputation-based methods often fail to fully exploit the complementary information of both time and frequency domains and remain vulnerable to distribution shifts [18].

In recent years, diffusion models [13,19], as powerful generative models, have achieved great success in fields such as Computer Vision (CV) and Audio Synthesis. Their iterative denoising mechanism offers a novel paradigm for time series modeling. CSDI [13] demonstrated the exceptional performance of diffusion models in time series imputation tasks. The imputation task requires the model to infer missing values based on contextual information, which is more deterministic than overall reconstruction or long-term prediction because it can utilize more conditional information (adjacent observations). Inspired by this, our paper explores the use of the imputation paradigm for anomaly detection, achieving significant results by intentionally masking parts of the data and calculating imputation errors to identify anomalies. Meanwhile, traditional anomaly detection methods are prone to anomaly bias and distribution shift. To mitigate these issues, temporal-frequency masked autoencoders combined with contrastive learning have been investigated. However, such methods rely on autoencoder-based reconstruction for contrastive learning and fail to take advantage of the stronger generative capacity and uncertainty modeling ability of diffusion models.

In light of this, this paper proposes the TFCID model, a Temporal-Frequency Contrastive Imputation Diffusion model. The core contribution lies in constructing a temporal-frequency dual-stream, contrastive-driven imputation diffusion framework. Specifically, in the temporal stream, we adopt an unconditional imputation diffusion model to reduce anomaly bias and generate high-quality imputation results. In the frequency stream, we use amplitude-aware frequency-domain masking to learn clean frequency-domain representations through a Transformer autoencoder. Most crucially, we design an adversarial contrastive learning module that no longer relies solely on reconstruction or imputation errors, but detects anomalies by measuring the discrepancy between the temporal and frequency representations of each point. This discrepancy is insensitive to distribution shift because the temporal-frequency representations of the same normal data should inherently be consistent, while anomalies disrupt this consistency.

The main contributions of this paper are as follows:

- Proposed a novel TFCID framework that integrates diffusion models, imputation, temporal-frequency analysis, and contrastive learning into a unified model framework, providing a new and powerful solution for multivariate time series anomaly detection.
- Designed an adversarial temporal-frequency contrastive learning mechanism for anomaly detection, which effectively mitigates the distribution shift problem by maximizing the distinguishability of representation discrepancies between normal and anomalous samples, thereby enhancing the model’s generalization ability and robustness.
- Employed innovative masking strategies in both the temporal and frequency domains. This effective masking strategy transforms potentially anomalous data into learnable latent space variables, thereby recovering variable features from the correlations of normal samples through diffusion models and neural network architectures.
- Extensive experiments conducted on multiple public datasets demonstrate that TFCID surpasses current mainstream methods in terms of detection accuracy and overall performance.

The remainder of this paper is structured as follows: Section 2 provides a systematic review of related research literature, outlining the current progress in the field. Section 3 formally defines the research problem and elaborates in detail on the technical specifics and implementation mechanisms of the proposed TFCID method. Section 4 systematically introduces the experimental design and evaluation methodology, comprehensively presents the experimental results, and conducts an in-depth ablation analysis. Section 5 summarizes the overall work, highlights the research conclusions, and offers prospects for future research directions.

## 2. Related Work

### 2.1. Time Series Anomaly Detection

Time series anomaly detection methods have evolved over many years, progressing from traditional statistical methods [20], distance-based methods [21], isolation-based methods [22] to the current mainstream deep learning methods [23]. Deep learning methods are highly favored for their ability to automatically learn complex nonlinear features and dependencies. These can be further subdivided into several categories:

**Reconstruction-based methods:** These methods assume that the model can only learn reconstruction patterns from normal data. Therefore, reconstruction errors for anomalous data will be larger. Early work, such as OmniAnomaly [1], combined VAE and GRU to learn robust representations. MAD-GAN [24] utilized the GAN discriminator loss as an additional anomaly indicator. In recent years, the Transformer architecture has been widely applied. For example, TranAD [25] uses attention mechanisms combined with adversarial training to improve detection accuracy. However, an inherent risk of reconstruction models is the potential for “over-generalization”, where an overly powerful model might reconstruct anomalous points reasonably well, leading to missed detections.

**Prediction-based methods:** These methods treat anomaly detection as a sequence prediction problem. For instance, LSTM-AD [10] uses LSTM to predict future values and uses prediction deviation as the anomaly score. MTAD-GAT [24] employs graph attention networks to simultaneously capture feature and temporal correlations. The challenge for prediction methods lies in the inherent uncertainty of future values. Accurate prediction is particularly difficult in complex systems, which may affect the stability of anomaly detection.

**Imputation-based methods:** Unlike reconstructing the entire sequence or predicting the future, imputation methods aim to fill randomly missing parts within a sequence. SAITS [12] is a pioneering work in this area based on the Transformer architecture, which effectively captures global dependencies in time series for accurate imputation using self-attention mechanisms. Research shows that using imputation errors for anomaly detection can be more accurate than reconstruction and prediction methods. Its advantage lies in the fact that the imputation task can utilize more neighboring contextual information as conditions, reducing prediction uncertainty. This work directly inherits and extends this idea.

### 2.2. Diffusion Models in Time Series Applications

Diffusion models [13], through a process of forward noising and reverse denoising, have demonstrated potential to surpass GAN [26] and VAE [27] in generation quality. In the field of time series, their applications mainly focus on generation [28], imputation [29], and prediction [30]. For example, CSDI [13] was the first to apply probabilistic diffusion models to time series imputation. TimeGrad [31] applies diffusion models in an autoregressive manner for future sequence prediction. In the area of anomaly detection, works like DiffusionAD [32] have applied diffusion models to image anomaly detection. However, research on introducing frequency domain information and contrastive learning into the diffusion model framework for multivariate time series anomaly detection is still limited. TFCID represents a novel attempt in this direction. The work in this paper further introduces frequency domain information and contrastive learning into the diffusion model framework based on this foundation.

Despite the success of diffusion models in time series imputation [13], a critical research gap remains in clearly delineating the boundary between imputation and anomaly detection. Imputation tasks aim to recover missing values by leveraging contextual information, assuming that the underlying data distribution is stationary and that missing patterns are random. In contrast, anomaly detection requires identifying samples that deviate from the learned normal distribution, where the “deviations” themselves may contaminate the training data. Existing diffusion-based imputation methods [13,29] primarily focus on accurate value recovery and do not explicitly address the risk of anomaly contamination during training. This distinction is crucial: when imputation models are directly applied to anomaly detection, they may inadvertently learn to reconstruct anomalies as if they were normal patterns, especially when the training data contains undetected anomalies. (4) Versus Iterative Feedback-based Diffusion Models [33]: This method incorporates an iterative feedback mechanism to refine detection results, demonstrating the potential of diffusion models in complex anomaly scenarios. However, it remains within a single-view (time-only) framework. TFCID further distinguishes itself by integrating frequency-domain analysis and adversarial contrastive learning to capture complementary anomaly patterns. TFCID uniquely combines unconditional diffusion imputation, frequency-domain amplitude masking, and adversarial contrastive learning to achieve robust anomaly detection under distribution shift.

Compared to existing state-of-the-art anomaly detection methods, TFCID introduces three key innovations that address their respective limitations. (1) Versus ImDiffusion [34]: While ImDiffusion pioneered the combination of imputation and diffusion models for anomaly detection, it relies on a conditional diffusion formulation where the model conditions on observed values during training. This conditional design may inadvertently propagate anomalous patterns into the learned distribution. In contrast, TFCID adopts an unconditional diffusion design with grating masking, which prevents direct exposure to potentially anomalous data. (2) Versus Conditional Weight-Incremental Diffusion Models [35]: This method addresses non-stationarity through weight-incremental mechanisms but remains within a single-domain (time-only) modeling paradigm. TFCID introduces a dual-stream temporal-frequency architecture that captures anomalies from complementary perspectives, enabling detection of both point anomalies and pattern-level periodicity disruptions. (3) Versus Latent Space Imputation Methods [36]: While performing imputation in latent space improves robustness to high-frequency fluctuations, these methods do not leverage the generative power of diffusion models nor the discriminative power of contrastive learning. TFCID uniquely combines unconditional diffusion imputation, frequency-domain amplitude masking, and adversarial contrastive learning to achieve robust anomaly detection under distribution shift.

### 2.3. Contrastive Learning and Temporal-Frequency Analysis

Contrastive learning [37] learns effective representations by pulling positive sample pairs closer and pushing negative sample pairs apart, achieving great success in unsupervised learning. In time series anomaly detection, works such as AnoTran [38] and DCdetector [39] have attempted to use contrastive learning to enhance model performance. The classic multi-view contrastive learning framework CMC [40] provides a core insight for solving the distribution shift problem in anomaly detection: the representations of the same data under different views should tend to be consistent. This paper is deeply inspired by this idea and applies it for the first time to the temporal and frequency views of time series. The difference is that we replace the traditional autoencoder reconstruction path with a more powerful diffusion model and introduce adversarial training to strengthen the effect of contrastive learning, forming a hybrid model with superior performance.

### 2.4. Transformer-Based Time Series Analysis

Transformer [41] was first applied in the field of Natural Language Processing (NLP). Owing to its powerful capability in processing sequential data, it has achieved great success in many other fields, such as audio processing, NLP, and CV [42,43]. Since its self-attention mechanism can effectively handle long-range dependencies in sequence data, it has been widely applied in time series analysis in recent years. Anomaly Transformer [44], TranAD [25], AnomalyBert [45], and others have extensively explored the application of Transformer [41] in anomaly detection methods. MEMTO [46] uses the Transformer [47] encoder for feature extraction and proposes a module incorporating memory gating, aiming to address detection errors in reconstruction-based models. These modeling methods typically embed multivariate data in a time-step manner. While this approach can capture temporal dependencies, it appears insufficient for modeling feature correlations between variables. In contrast, this paper employs an inverted embedding strategy when extracting features in the frequency domain to enhance the model’s ability to capture complex interactions between variables, while simultaneously combining a multivariate memory unit approach to more precisely capture the personalized characteristics of multivariate data.

### 2.5. Domain Generalization for Anomaly Detection

Recent advances in domain generalization have explored feature decoupling techniques to handle distribution shifts in fault diagnosis [48,49]. Specifically, domain feature decoupling networks [48] separate domain-related features from fault-related features, enabling robust diagnosis under unseen operating conditions. Similarly, joint collaborative adaptation networks address class imbalance and variable operating conditions through adaptive feature alignment. While these methods primarily focus on supervised fault diagnosis tasks, their core insight—that disentangling domain-invariant and domain-specific features enhances generalization—informs our adversarial contrastive learning design. TFCID extends this idea to unsupervised anomaly detection by learning temporal and frequency representations that are mutually consistent for normal data but diverge for anomalies, effectively mitigating distribution shift without requiring labeled data.

## 3. Methodology

### 3.1. Problem Definition

For the unsupervised time series anomaly detection (TSAD) task, the training set consists solely of unlabeled multivariate data, denoted as $D_{\mathrm{train}}=\left\{x_{1},x_{2},\ldots ,x_{L_{\mathrm{train}}}\right\}$, where each $x_{t}\in R^{K}$ is a *K*-dimensional vector. The test set includes test data $D_{\mathrm{test}}=\left\{x_{1},x_{2},\ldots ,x_{L_{\mathrm{test}}}\right\}$ and the corresponding true anomaly labels $Y=\{y_{1},y_{2},\ldots ,y_{L_{\mathrm{test}}}\}$, where $y_{t}\in \left\{0,1\right\}$ ($y_{t}=1$ indicates that time point *t* is anomalous). The labels correspond one-to-one with the time series data at each timestamp in the test set, indicating whether the data at that timestamp is abnormal. During the training phase, the model captures the distribution characteristics of normal data through self-supervised learning, avoiding the use of any label information. During the inference process, the model calculates an anomaly score $x_{t}$ for each time point and determines an anomaly through a threshold θ:

(1) $${\hat{y}}_{t}=\left\{\begin{array}{cc} 1, & \mathrm{if}\;Score(x_{t})\geq \theta \\ 0, & \mathrm{if}\;Score(x_{t})<\theta \end{array}\right.$$

The optimization goal of the anomaly detection algorithm is to make the predicted labels $\hat{Y}$ as close as possible to the ground truth Y. TFCID aims to solve the problems of anomaly bias and distribution shift in traditional methods by integrating temporal-domain imputation diffusion models and frequency-domain masked autoencoders with a temporal-frequency contrastive learning framework, thereby improving detection accuracy and robustness.

### 3.2. Model Overview

**Figure 1:**
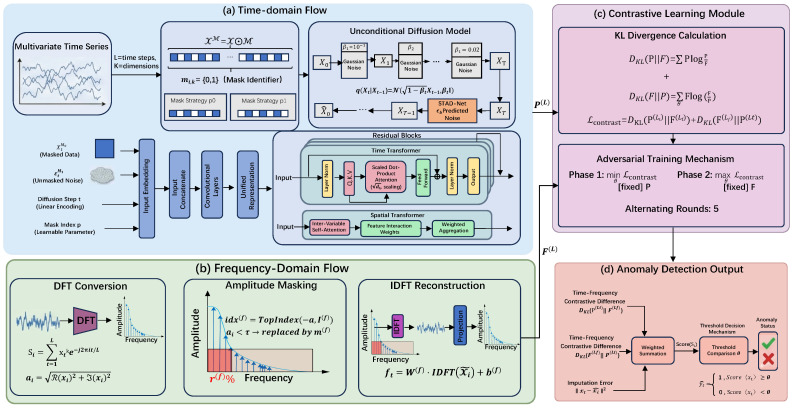
The overall framework of TFCID: The model synergizes temporal diffusion reconstruction with a frequency-domain amplitude masking mechanism through a contrastive learning module, aiming to capture the time-frequency characteristics of multivariate time series and achieve anomaly detection.

TFCID (Temporal-Frequency Contrastive Imputation Diffusion Model) is an innovative dual-stream architecture model that achieves efficient time series anomaly detection through collaborative processing of temporal and frequency domain paths, combined with a contrastive learning mechanism. As shown in Figure 1, the model mainly consists of four components:

(1) **Temporal Processing Stream**: Uses a grating masking strategy and an unconditional diffusion model for time series imputation, employing a residual Transformer (STAD-Net) to learn temporal dependencies.

(2) **Frequency Processing Stream**: Applies amplitude-based frequency-domain masking and a Transformer autoencoder to learn frequency-domain patterns.

(3) **Contrastive Learning Module**: Computes the discrepancy between temporal and frequency representations as an anomaly signal.

(4) **Adversarial Training Module**: Prevents overfitting by maximizing temporal-frequency discrepancies.

During training, the input MTS (Multivariate Time Series) data is respectively masked in the temporal and frequency domains to generate two views. The temporal stream imputes the masked values via the diffusion model, the frequency stream recovers the masked frequencies via the autoencoder, and finally, the temporal and frequency representations are aligned through a contrastive loss function. During inference, the anomaly score combines the imputation error and the contrastive discrepancy to achieve accurate detection. This architecture captures multi-dimensional anomaly patterns through temporal-frequency complementarity, while utilizing contrastive learning to mitigate distribution shift.

### 3.3. Temporal Processing Module

The temporal module combines a grid-based masking strategy with a diffusion model to perform accurate time series imputation. By leveraging structured masking together with a progressive denoising process, the module effectively captures temporal dependencies within the sequences while mitigating the influence of anomalous observations during model training.

#### 3.3.1. Grating Masking Strategy

**Figure 2:**
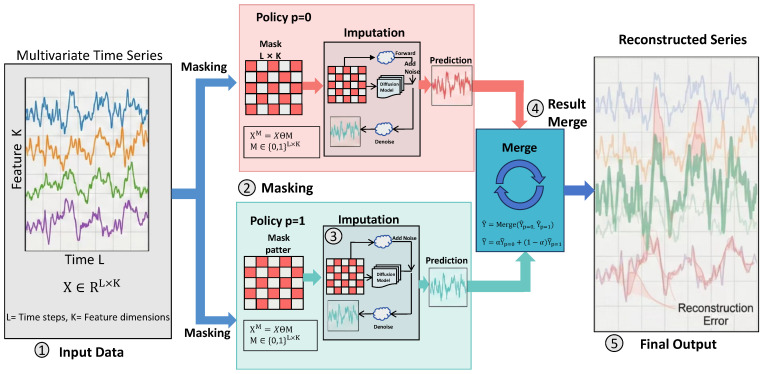
Temporal masking strategy: This illustrates the alternating masking of multivariate time series through two complementary masking strategies (p=0 and p=1), along with the merging of dual-path imputation predictions to achieve full sequence reconstruction.

The temporal masking adopts a Grating Masking strategy, as illustrated in Figure 2 and detailed in Algorithm 1. Given a multivariate time series input $X=\{x_{1},x_{2},\ldots ,x_{L}\}$, the mask tensor $M\in {\left\{0,1\right\}}^{L\times K}$ defines the masked positions, where $m_{l,k}=0$ indicates that position (l,k) is masked. Grating masking utilizes two complementary masking strategies (indexed by p∈{0,1}), ensuring that each data point is imputed. The masked data is represented as $X^{M}=X⊙M$ ($M=M_{0}+M_{1}$), where ⊙ denotes element-wise multiplication (Hadamard product), and $M_{0},M_{1}$ represent the two complementary binary mask tensors corresponding to the grating strategies (indexed by p∈{0,1}). The advantage of grating masking lies in its provision of partial contextual information: it both leverages adjacent observations to reduce uncertainty and partially “peeks” at future values to enhance timeliness. The mask index *p* is fed as a conditional input to the diffusion model, reducing ambiguity.

#### 3.3.2. Unconditional Diffusion Model Imputation

**Figure 3:**
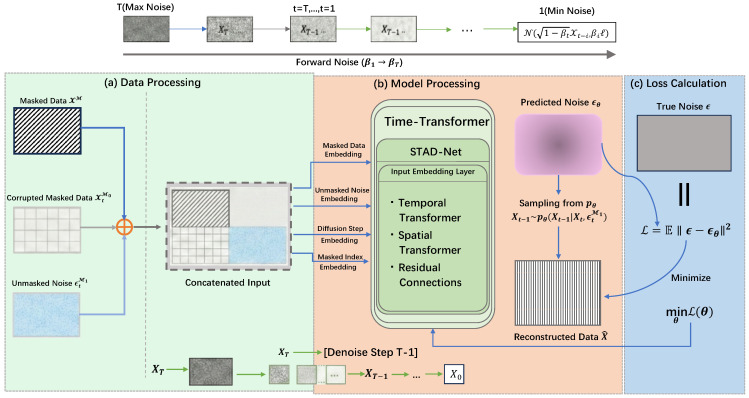
Unconditional Diffusion Model Interpolation: This figure illustrates the process of constructing inputs by concatenating masked data with noise. It utilizes a Time Transformer to extract spatiotemporal features for noise prediction, and finally achieves time series reconstruction through reverse iterative denoising.

TFCID employs an unconditional diffusion model for imputation, with the process shown in Figure 3. The diffusion model includes a forward noise-adding process and a reverse denoising process. The forward process gradually adds Gaussian noise to the complete data $X_{0}$:

(2) $$q\left(X_{t}|X_{t-1}\right)=N\left(X_{t};\sqrt{1-\beta_{t}}X_{t-1},\beta_{t}I\right)$$

where $\beta_{t}$ is the noise level at step *t*. After *T* steps, the data approximately becomes pure noise $X_{T}∼N\left(0,I\right)$. The reverse process uses a parameterized model Θ to denoise and progressively recover the data. For unconditional imputation, the model conditions on the masked portion $X_{t}^{M_{0}}$ and the forward noise of the unmasked portion $\epsilon_{t}^{M_{1}}$ to estimate the denoising function:

(3) $$\mu_{\Theta }\left(X_{t}^{M_{0}},t,\epsilon_{t}^{M_{1}}\right)=\tilde{\mu }\left(X_{t}^{M_{0}},t,\epsilon_{\Theta }\left(X_{t}^{M_{0}},t,\epsilon_{t}^{M_{1}}\right)\right)$$

where $\epsilon_{\Theta }$ is the noise predicted by the network. The training objective is to minimize the noise prediction error:

(4) $$\min_{\Theta }L_{\mathrm{diff}}=E_{X_{0}∼q\left(X_{0}\right),\epsilon ∼N\left(0,I\right),t}{∥\epsilon -\epsilon_{\Theta }\left(X_{t}^{M_{0}},t,\epsilon_{t}^{M_{1}}\right)∥}^{2}$$

The unconditional design ensures that the model indirectly references the unmasked data through noise, avoiding the leakage of anomalous values and enhancing the decision boundary for anomaly detection.

**Algorithm 1** Grating Masking Strategy
**Require:** Multivariate time series $X\in R^{L\times K}$, block window size *w*, mask ratio $r^{\left(T\right)}$  1:Calculate the number of partitions: N←⌈L/(2·w)⌉  2:Partition the time steps into 2×N consecutive blocks of size *w*  3:**for** mask index p∈{0,1} **do**  4:    **if** p=0 **then**  5:        Mask the first *w* time steps of each block pair  6:        Keep the remaining *w* time steps unmasked to form mask matrix $M_{0}$  7:    **else**                        ▹ Complementary pattern  8:        Keep the first *w* time steps unmasked  9:        Mask the remaining *w* time steps to form mask matrix $M_{1}$10:    **end if**11:
**end for**
12:**Verify constraint:** $M_{0}+M_{1}=1$ (element-wise)**Ensure:** Mask matrices $M_{0},M_{1}$


Crucially, the effectiveness of the unconditional diffusion model in capturing temporal dependencies stems from the synergy between the grating masking strategy and the iterative denoising process. The grating masking partitions the input into two complementary views, forcing the model to infer masked values from context without directly observing the original data. During reverse diffusion, the model must progressively reconstruct the complete sequence by learning the underlying temporal correlations across time steps. This process compels the model to internalize both short-term autocorrelations and long-range dependencies, enabling high-quality imputation that remains robust to anomalous observations.

Methodological Novelty: The proposed diffusion model differs from existing diffusion-based imputation methods (e.g., CSDI [13]) in two fundamental aspects. First, unconditional design: while CSDI conditions on observed values during training, which may inadvertently propagate anomalous patterns into the learned distribution, our model adopts an unconditional formulation that leverages grating masking to prevent direct exposure to potentially anomalous data. This forces the model to learn the underlying normal data distribution without being biased by anomalies. Second, architectural integration: unlike prior works that treat diffusion models as standalone imputation tools, our diffusion model is embedded within a larger temporal-frequency contrastive learning framework. Its output serves not only for imputation but also as a temporal representation that is explicitly contrasted with frequency-domain representations. This synergistic design enables the detection of anomalies that manifest as inconsistencies between temporal and frequency views, which is a novel capability absent in existing diffusion-based anomaly detection approaches.

#### 3.3.3. Theoretical Justification for Unconditional Diffusion Design

The superiority of unconditional diffusion over conditional diffusion for anomaly detection can be justified from two perspectives: **anomaly contamination avoidance** and **distribution boundary learning**.

1. Anomaly Contamination Avoidance:

Conditional diffusion models, such as CSDI [13] and ImDiffusion [34], are trained to learn $p_{\theta }\left(X_{0}|X_{\mathrm{cond}}\right)$, where the condition $X_{\mathrm{cond}}$ is typically the observed data. When the training set contains undetected anomalies, the model learns to reconstruct anomalies as if they were normal patterns because the condition itself carries anomalous information. Formally, let $D_{\mathrm{train}}$ contain a mixture of normal samples N and anomalous samples A. A conditional model minimizes

$$E_{x∼N\cup A}\left[∥\epsilon -\epsilon_{\theta }\left(x_{t},t,x_{\mathrm{cond}}\right){∥}^{2}\right],$$

which encourages the model to fit both normal and anomalous distributions. In contrast, our unconditional design with grating masking ensures that the model never directly observes the original data as a condition. The reverse process conditions only on the masked portion $X_{t}^{M_{0}}$ and noise $\epsilon_{t}^{M_{1}}$, forcing the model to infer the complete data distribution from contextual information without being biased by potential anomalies. This aligns with recent findings that unconditional models can achieve comparable or even superior performance when properly conditioned on self-supervised features [50].

2. Distribution Boundary Learning:

From a decision boundary perspective, anomaly detection requires a clear separation between the normal distribution and the anomalous region. Conditional models, by fitting the conditional distribution $p(X_{0}|X_{\mathrm{cond}})$, tend to produce sharp reconstructions that may inadvertently span the anomaly region when the condition is anomalous. Unconditional models, by contrast, learn the marginal distribution $p(X_{0})$ and generate samples through iterative denoising that inherently respects the geometry of the normal data manifold. As demonstrated in recent work [50], unconditional guidance can provide disentangled control over generation quality, avoiding the overfitting issues associated with conditional guidance. In our framework, the adversarial contrastive learning further amplifies the discrepancy between temporal and frequency representations for anomalous samples, leveraging the clean boundary established by the unconditional diffusion backbone.

#### 3.3.4. STAD-Net Architecture

**Figure 4:**
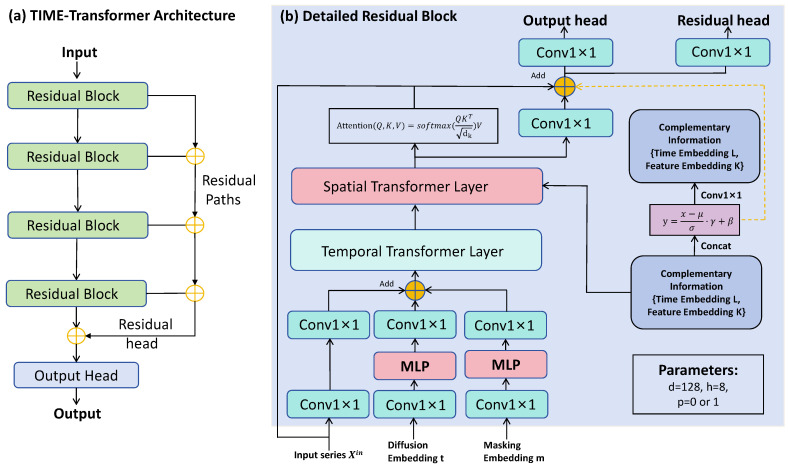
The STAD-Net architecture consists of multiple stacked residual blocks, each containing spatio-temporal Transformer layers and auxiliary embedding modules. It is designed to effectively capture multi-dimensional spatio-temporal dependencies through deep residual connections.

The imputation process uses a dedicated STAD-Net model, whose architecture is shown in Figure 4.

This is a Transformer network based on a residual structure, used to capture the temporal and feature-dimensional dependencies of MTS. The input to STAD-Net consists of five parts: (1) Masked data $X_{t}^{M_{0}}$. (2) Unmasked noise $\epsilon_{t}^{M_{1}}$ (known during training, used as a condition). (3) Embedding of the diffusion step *t*; (4) Embedding of the mask index *p*; (5) Positional encodings for the time and feature dimensions.

These inputs are projected to a unified dimension through convolutional or fully connected layers and then fed into residual blocks. Each residual block contains a temporal Transformer layer and a spatial Transformer layer: the temporal Transformer weights features at different time steps through self-attention, dynamically handling the mask state; the spatial Transformer captures inter-variable correlations. The output is fused with the input through residual connections, enhancing gradient propagation.

**Key innovations of STAD-Net compared to standard Transformers:** (1) **Dual-stream spatiotemporal attention:** Unlike standard Transformers that apply self-attention only along the temporal dimension, STAD-Net explicitly separates temporal and spatial attention into two distinct layers. The temporal Transformer models dependencies across time steps, while the spatial Transformer captures correlations across different variables at the same time step. This decoupling reduces computational complexity from $O({\left(L\cdot K\right)}^{2})$ to $O(L^{2}+K^{2})$ per layer, where *L* is the sequence length and *K* is the number of variables, while preserving the ability to model both dimensions. (2) **Mask-aware attention mechanism:** Standard Transformers treat all positions uniformly, which is suboptimal for imputation tasks where masked positions require different treatment. STAD-Net incorporates the mask index *p* as a conditional input, enabling the attention mechanism to dynamically adjust its focus based on which positions are masked. This allows the model to better exploit observed contextual information when inferring missing values. (3) **Diffusion step conditioning:** Unlike conventional Transformers that operate on static inputs, STAD-Net receives the diffusion step *t* as an additional embedding. This allows the network to adapt its behavior according to the noise level at different stages of the reverse diffusion process, improving the quality of iterative denoising.

The mathematical expression of STAD-Net is:

(5) $$\epsilon_{\Theta }=\mathrm{STAD}\mathrm{-Net}\left(X_{t}^{M_{0}},t,\epsilon_{t}^{M_{1}},p\right).$$

This design accurately models the complex dependencies in MTS through a hierarchical attention mechanism, improving the quality of imputation.

### 3.4. Frequency Domain Processing Module

The frequency-domain module operates on transformed spectral representations and incorporates an amplitude-aware masking mechanism together with an autoencoder-based reconstruction framework. This design focuses on modeling periodic and trend-related characteristics in time series, thereby enhancing the detection of pattern-level anomalies. This module complements the temporal processing stream, jointly building a complete anomaly detection system. The overall framework of the frequency module is shown in Figure 1b Frequency-Domain Flow.

This amplitude-aware masking strategy enables the Transformer autoencoder to learn clean frequency-domain representations. By masking small-magnitude frequency components—which are likely to contain noise or transient anomalies—the model is forced to rely on dominant frequency components that capture the core periodic and trend patterns during reconstruction. Furthermore, the self-attention mechanism in the Transformer encoder models global dependencies across different frequency bands, allowing the network to capture complex interactions between periodic patterns and learn a compact, noise-resilient latent representation.

#### 3.4.1. Amplitude-Based Frequency Domain Masking

Frequency-domain masking is applied directly to the spectral representation of the input time series to capture structural pattern anomalies. First, the raw multivariate time series data $D_{\mathrm{input}}$ is transformed into its frequency-domain representation $S\in C^{|L|\times K}$ via the Discrete Fourier Transform (DFT):

(6) $$s_{i}^{k}=\sum_{t=1}^{\left|L\right|}\left(x_{t}^{k}e^{-j\omega_{i}^{k}t}\right),$$

where $x_{t}^{k}$ denotes the value of the *k*-th feature at time *t* within the time series; *e* is the base of the natural logarithm (Euler’s number, e≈2.71828); *j* represents the imaginary unit ($j^{2}=-1$); $\omega_{i}^{k}=\frac{2\pi i}{\left|L\right|}$ is the angular frequency for feature *k*; *L* is the length of the time series; *i* is the frequency index (i=0,1,…,L−1); and *k* is the feature index (k=1,2,…,K). Subsequently, the magnitude $A\in R^{L\times K}$ is computed as follows:

(7) $$a_{i}^{k}=\sqrt{R{\left(x_{i}^{k}\right)}^{2}+J{\left(x_{i}^{k}\right)}^{2}},$$

where $R(s_{i}^{k})$ denotes the real part of the complex number $s_{i}^{k}$, and $J(s_{i}^{k})$ represents its imaginary part; $a_{i}^{k}$ signifies the magnitude of the *k*-th feature at the *i*-th frequency component.

Frequency components with small magnitudes may correspond to transient or anomalous patterns. The $r^{\left(F\right)}\%$ of frequencies with the smallest magnitudes are selected for masking ($r^{\left(F\right)}$ is a hyperparameter), with the mask indices $idx^{\left(F\right)}$ determined by the TopIndex function. The masked frequencies are replaced with a learnable vector $m^{\left(F\right)}$, generating an updated frequency domain representation $\tilde{X}$. Finally, $\tilde{X}$ is converted back to the time domain via the Inverse DFT (IDFT) and linearly projected into a latent space. Amplitude-based masking avoids the limitations of traditional high-frequency filtering, allowing for a more comprehensive assessment of pattern importance.

#### 3.4.2. Theoretical Justification for Magnitude-Based Frequency Masking

The selection of low-magnitude frequency components as masking targets is grounded in the **energy compaction property** of the Fourier transform for real-world time series. Dominant periodic and trend patterns are typically concentrated in a few high-magnitude frequency bins, whereas sensor noise, high-frequency fluctuations, and transient artifacts are distributed across a large number of low-magnitude components [51,52]. Masking these low-energy components minimizes the impact on reconstruction fidelity (as measured by MSE) while effectively suppressing noise, thereby forcing the autoencoder to prioritize the most informative frequency bands for representation learning [51].

#### 3.4.3. Addressing the Risk of Removing Subtle Signals

We acknowledge the potential concern that critical but subtle signals (e.g., incipient fault signatures) may reside in low-magnitude frequency bands. However, our masking strategy incorporates three key safeguards against this risk:

1. **Adaptive Thresholding**: The masking ratio $r^{\left(F\right)}\in \left[10\%,40\%\right]$ is a hyperparameter that is optimized per dataset. In scenarios where subtle signals are known to be critical (e.g., low signal-to-noise ratio environments), the model can automatically converge to a lower $r^{\left(F\right)}$ value to preserve a wider frequency band [51].
2. **Learnable Recovery**: Crucially, masked components are not simply discarded but are replaced by a learnable vector $m^{\left(F\right)}$. This allows the model to recover the contribution of any erroneously masked but informative frequency component during training, transforming the operation from a hard deletion to a soft, data-dependent gating mechanism [51,53].
3. **Empirical Priority**: In anomaly detection, the primary objective is to capture gross deviations in dominant patterns. Over-preserving low-magnitude components to protect against hypothetical subtle faults often leads to increased false positives due to noise amplification [52]. Our approach strikes a balance by prioritizing robustness to common anomalies.

#### 3.4.4. Transformer Autoencoder

The frequency domain view uses a Transformer-based autoencoder for representation learning. The self-attention mechanism in the Transformer encoder plays a crucial role in integrating global frequency-domain information. Unlike time-domain processing, where dependencies are primarily local and sequential, frequency-domain representations encode periodic patterns across the entire sequence. The self-attention mechanism allows the model to capture complex interactions between different frequency components, such as harmonic relationships and cross-frequency dependencies, enabling the learning of a holistic and compact representation that preserves essential periodic structures while filtering out noise. Due to the mixing of frequency domain data after the inverse transform, a decoder-only architecture is adopted. The input frequency-masked data first has sequential information added via sinusoidal positional encoding:

(8) $$f_{t}^{\left(0\right)}=f_{t}+c_{t},$$

among these, $f_{t}$ denotes the input frequency-domain masked data at the *t*-th position, $c_{t}$ is the positional encoding vector at the *t*-th position, $f_{t}^{\left(0\right)}$ represents the embedding at the *t*-th position in the 0-th layer (input layer), *t* is the position index (t=1,2,…,T), and *T* is the sequence length. Subsequently, *L* layers of Transformer modules process the data: each layer includes a multi-head self-attention mechanism and a feed-forward network. The self-attention computes scaled dot-product attention for queries (*Q*), keys (*K*), and values (*V*):

(9) $${\tilde{F}}^{\left(l\right)}=\mathrm{Softmax}\left(\frac{Q^{\left(l\right)}K^{(l)⊤}}{\sqrt{D}}\right)V^{\left(l\right)},$$

where *l* denotes the Transformer layer index (l=1,2,…,L), $Q^{\left(l\right)}=F^{\left(l-1\right)}W_{Q}^{\left(l\right)}$ is the query matrix of the *l*-th layer, $K^{\left(l\right)}=F^{\left(l-1\right)}W_{K}^{\left(l\right)}$ is the key matrix of the *l*-th layer, $V^{\left(l\right)}=F^{\left(l-1\right)}W_{V}^{\left(l\right)}$ is the value matrix of the *l*-th layer, $W_{Q}^{\left(l\right)},W_{K}^{\left(l\right)},W_{V}^{\left(l\right)}$ are learnable weight matrices, *D* is the dimension of the attention mechanism (used for scaling the dot product), ⊤ denotes matrix transpose operation, and ${\tilde{F}}^{\left(l\right)}$ is the output of the self-attention mechanism at the *l*-th layer. The representation is further optimized through residual connections and layer normalization:

(10) $$F^{\left(l\right)}=\mathrm{LayerNorm}\left({\tilde{F}}^{\left(l\right)}+\mathrm{MLP}\left({\tilde{F}}^{\left(l\right)}\right)\right),$$

where MLP(·) denotes the multilayer perceptron (feed-forward network), LayerNorm(·) denotes the layer normalization operation, and $F^{\left(l\right)}$ is the final output of the *l*-th Transformer module. The autoencoder outputs the frequency domain representation $F^{\left(L\right)}$, which is used for subsequent contrastive learning. This design effectively captures long-term dependencies in frequency domain patterns.

### 3.5. Contrastive Learning and Adversarial Training

The core innovation of TFCID is to replace traditional reconstruction errors with temporal-frequency contrastive learning to solve the distribution shift problem, combined with adversarial training to enhance robustness. Its framework is shown in Figure 1c Contrastive Learning Module.

#### 3.5.1. Temporal-Frequency Contrastive Learning

Contrastive learning aims to minimize the discrepancy between the temporal representation $P^{\left(L\right)}$ (output from STAD-Net) and the frequency representation $F^{\left(L\right)}$ (output from the frequency domain autoencoder). Since the temporal and frequency views correspond to the same time series, their representations should be consistent, while anomalous data will cause a large discrepancy. The Kullback-Leibler divergence (KLD) is used as the discrepancy measure:

(11) $$L_{\mathrm{contrast}}=D_{\mathrm{KL}}\left(P^{\left(L_{t}\right)}∥F^{\left(L_{f}\right)}\right)+D_{\mathrm{KL}}\left(F^{\left(L_{f}\right)}∥P^{\left(L_{t}\right)}\right),$$

where $D_{\mathrm{KL}}\left(P∥Q\right)=\sum_{i}P_{i}\log\left(P_{i}/Q_{i}\right)$. This loss function encourages the alignment of temporal and frequency representations for normal data, while anomalous data produces high discrepancies due to misalignment. Contrastive learning is distribution-agnostic, effectively mitigating the impact of distribution shift between training and test data.

#### 3.5.2. Normalization for Distribution Representation

Before computing the KL divergence, the temporal representation $P(L_{t})$ and frequency representation $F(L_{f})$ must be converted into valid probability distributions. Following standard practice, we apply the softmax function along the feature dimension:

(12) $${\tilde{P}}_{i,j}=\frac{\exp(P_{i,j}/\tau )}{\sum_{k=1}^{D}\exp\left(P_{i,k}/\tau \right)},\;{\tilde{F}}_{i,j}=\frac{\exp(F_{i,j}/\tau )}{\sum_{k=1}^{D}\exp\left(F_{i,k}/\tau \right)}$$

where *D* is the representation dimension and τ is a temperature hyperparameter (set to 1.0 in our experiments). This normalization ensures that for each time step, $\sum_{i}{\tilde{P}}_{i,j}=1$ and ${\tilde{P}}_{i,j}\geq 0$ (similarly for $\tilde{F}$), satisfying the fundamental requirements of a probability distribution and enabling meaningful KL divergence computation.

#### 3.5.3. Adversarial Training Mechanism

To prevent overfitting in contrastive learning and enhance the model’s ability to distinguish between normal and anomalous data, we introduce adversarial training. Specifically, this is achieved by alternately optimizing the following two objectives:

- **Minimization Phase (Update Frequency Encoder)**: Fix the temporal representation $P^{\left(L_{t}\right)}$, update the parameters of the frequency encoder to minimize $L_{\mathrm{contrast}}$, causing the frequency representation to move closer to the temporal representation.
- **Maximization Phase (Update Temporal Encoder)**: Fix the frequency representation $F^{\left(L_{f}\right)}$, update the parameters of the temporal encoder to maximize $L_{\mathrm{contrast}}$, causing the temporal representation to move away from the current (potentially unoptimized) frequency representation. This step is implemented via a Gradient Reversal Layer (GRL).

The adversarial objective can be formalized as:

(13) $$L_{\mathrm{adv}}=\min_{\Theta_{F}}\max_{\Theta_{P}}\left(D_{\mathrm{KL}}\left(P^{\left(L_{t}\right)}∥F^{\left(L_{f}\right)}\right)+D_{\mathrm{KL}}\left(F^{\left(L_{f}\right)}∥P^{\left(L_{t}\right)}\right)\right),$$

where $\Theta_{P}$ and $\Theta_{F}$ are the parameters of the temporal and frequency encoders, respectively. Adversarial training ensures that the model learns more robust representations: the temporal-frequency representations of normal data tend to converge during the adversarial process, while the discrepancies for anomalous data are amplified.

##### Relationship Between Adversarial Training and Contrastive Learning

The contrastive objective $L_{\mathrm{contrast}}$ encourages alignment between temporal and frequency representations. However, without adversarial training, this objective can lead to trivial solutions where both encoders simply collapse to the same constant output. The adversarial training mechanism prevents this collapse by introducing a competition: the frequency encoder is updated to minimize the KL divergence (moving closer to temporal representations), while the temporal encoder is updated to maximize the KL divergence (moving away from frequency representations). This minimax game ensures that both encoders learn informative, discriminative features rather than trivial alignments.

##### Convergence Behavior

We employ an alternating optimization strategy with 100 rounds per training epoch. In practice, we observe that the contrastive loss decreases steadily during the first 30–40 epochs and then stabilizes. The adversarial dynamics do not lead to oscillation or divergence because the gradient reversal layer introduces a fixed scaling factor λ, which empirically stabilizes training. Early stopping based on validation set performance prevents overfitting. The complementary roles of the two objectives—contrastive learning for alignment, adversarial training for discrimination—work synergistically to produce robust representations.

### 3.6. Objective Function

The total loss function of TFCID combines the temporal imputation loss and the temporal-frequency contrastive loss, optimized through adversarial training. The temporal imputation loss ensures that the diffusion model accurately predicts the masked values:

(14) $$L_{\mathrm{impute}}={∥X-\hat{X}∥}^{2},$$

where $\hat{X}$ is the imputation output of the diffusion model. The contrastive loss $L_{\mathrm{contrast}}$ is given by Equation (11) as described above. The total loss is:

(15) $$L_{\mathrm{total}}=L_{\mathrm{impute}}+\lambda L_{\mathrm{contrast}},$$

where λ is a hyperparameter that balances the imputation and contrastive terms. Adversarial training is implemented through alternating optimization of Equation (13) and is not directly incorporated into the total loss.

### 3.7. Anomaly Criterion

During inference, the anomaly score combines imputation error and time-frequency contrastive divergence. For each time point *t* in the test set $D_{\mathrm{test}}$, the score is computed as:

(16) $$\mathrm{Score}\left(x_{t}\right)=D_{\mathrm{KL}}\left(P_{t}^{\left(L_{t}\right)}∥F_{t}^{\left(L_{f}\right)}\right)+D_{\mathrm{KL}}\left(F_{t}^{\left(L_{f}\right)}∥P_{t}^{\left(L_{t}\right)}\right)+\gamma {∥x_{t}-{\hat{x}}_{t}∥}^{2},$$

among these, $P_{t}^{\mathrm{time}}$ and $F_{t}^{\mathrm{freq}}$ are the probability distributions obtained after normalizing the final representations output by the time-domain and frequency-domain encoders at time step *t*, respectively; ${\hat{x}}_{t}$ is the imputation reconstruction value of the model in the time domain. The first two terms in the formula constitute the symmetric KL divergence, which measures the consistency of temporal and frequency features in the semantic space, while the third term balances the magnitude difference between the reconstruction error and the divergence measure through a scaling factor γ>0. A higher $\mathrm{Score}(x_{t})$ value indicates that the observation at that time point deviates more from the normal pattern. Finally, binary anomaly labels ${\hat{y}}_{t}$ are generated using an optimized threshold θ on the validation set. This criterion integrates the deviation of feature distributions and reconstruction residuals in the physical space, enabling the simultaneous detection of pattern anomalies (e.g., periodic disruptions) and point anomalies (e.g., instantaneous spikes).

### 3.8. Training and Inference Algorithms

The training and inference processes of TFCID are detailed in Algorithms 2 and 3, respectively.

TFCID adopts a dual-stream temporal–frequency modeling framework to jointly capture multi-dimensional anomaly characteristics and further integrates contrastive learning with adversarial training. Experimental results on multiple benchmark datasets demonstrate that the proposed method achieves state-of-the-art detection performance. Beyond accuracy improvements, the integration of imputation and contrastive signals also enhances the robustness of the model in complex scenarios.

**Algorithm 2** TFCID Training Algorithm
**Require:** Training data $D_{train}$, grating mask ratio $r^{\left(T\right)}$, frequency domain mask ratio $r^{\left(F\right)}$, diffusion steps *T*, hyperparameter λ  1:Initialize model parameters  2:**for** each epoch **do**  3:    **for** each batch of data X **do**  4:        **Temporal Processing Stream:**  5:        Apply grating masking to generate masked data $X^{M}$  6:        Forward diffusion: Add noise to the masked part, obtaining $X_{t}^{M_{0}}$  7:        Reverse diffusion: Use STAD-Net to predict noise, calculate imputation loss $L_{\mathrm{impute}}$  8:        Output temporal representation $P^{\left(L\right)}$  9:        **Frequency Processing Stream:**10:        Apply amplitude-based frequency domain masking, generate frequency domain representation $\tilde{X}$11:        Process through Transformer autoencoder, output frequency domain representation $F^{\left(L\right)}$12:        Calculate contrastive loss $L_{\mathrm{contrast}}=D_{KL}\left(P^{\left(L\right)}∥F^{\left(L\right)}\right)+D_{KL}\left(F^{\left(L\right)}∥P^{\left(L\right)}\right)$13:        **Adversarial Training:**14:        Minimization phase: Fix $P^{\left(L\right)}$, update frequency domain parameters to minimize $L_{\mathrm{contrast}}$15:        Maximization phase: Fix $F^{\left(L\right)}$, update temporal domain parameters to maximize $L_{\mathrm{contrast}}$16:        Total loss $L_{\mathrm{total}}=L_{\mathrm{impute}}+\lambda L_{\mathrm{contrast}}$17:        Backpropagate to update all parameters18:    **end for**19:
**end for**
**Ensure:** Trained model


**Algorithm 3** TFCID Inference Algorithm
**Require:** Test data $D_{\mathrm{test}}$, trained model  1:**for** each time point *t* in $D_{\mathrm{test}}$ **do**  2:    Execute the temporal processing stream, obtain imputed value ${\hat{x}}_{t}$ and temporal representation $P_{t}^{\left(L\right)}$  3:    Execute the frequency processing stream, obtain frequency representation $f_{t}^{\left(L\right)}$  4:    Calculate anomaly score:  5:    $\mathrm{Score}\left(X_{t}\right)=D_{KL}\left(P_{t}^{\left(L\right)}∥F_{t}^{\left(L\right)}\right)+D_{KL}\left(F_{t}^{\left(L\right)}∥P_{t}^{\left(L\right)}\right)+{∥x_{t}-{\hat{x}}_{t}∥}^{2}$  6:
**end for**
  7:Generate anomaly labels $\hat{Y}$ based on the preset threshold θ**Ensure:** Anomaly labels $\hat{Y}$


## 4. Experiments

To comprehensively evaluate the performance of the TFCID model, we designed extensive experiments aimed at answering the following key research questions (RQ):

- RQ1: How does the performance of TFCID compare to current state-of-the-art anomaly detection methods?
- RQ2: What is the generalization capability of the model across different types of datasets (including different anomaly ratios, different sequence lengths, and different data distribution characteristics)?
- RQ3:Whether the model is effective in detecting different types of anomalies?
- RQ4: What is the contribution of each key component in the model (e.g., temporal-frequency contrastive learning, unconditional diffusion, adversarial training) to the final performance?
- RQ5: How sensitive is the model to different hyperparameters (e.g., masking ratio, number of Transformer layers)?

### 4.1. Experimental Setup

#### 4.1.1. Benchmark Datasets

We evaluated on five widely used public real-world datasets, which cover different domains and scales. The description of these datasets is as follows, and we summarize their characteristics in Table 1.

(1) SMD (Server Machine Dataset) [1]: Server machine metrics from a large internet company, containing 38-dimensional data from 28 servers. (2) PSM (Pooled Server Metrics [54]): The PSM dataset is a public dataset from eBay Server Machines, with 25 features describing server machine indicators (e.g., CPU utilization and memory). (3) SWaT (Secure Water Treatment [55]): This dataset was collected from a real-world water treatment plant, consisting of 51-dimensional sensor data collected from a continuously operating critical infrastructure system. (4) MSL (Mars Science Laboratory [3]): The MSL dataset is telemetry and engineering data collected by NASA’s Curiosity rover during scientific missions on the Martian surface, containing 55-dimensional sensor monitoring features, used for spacecraft system anomaly detection and fault diagnosis research. (5) SMAP (Soil Moisture Active Passive [3]): The SMAP dataset is data from NASA using soil samples and telemetry information from the Mars rover, with 25 features, used to study the spatiotemporal variations of soil moisture.

#### 4.1.2. Evaluation Metrics

To evaluate the performance of anomaly detection, we use Accuracy, Precision, Recall, and F1-Score to measure the model’s anomaly detection capability. The formulas for each metric are as follows:

(17) $$\begin{array}{cc} \mathrm{Precision} & =\frac{TP}{TP+FP}\;\;,\;\mathrm{Recall}=\frac{TP}{TP+FN}\;\;,\;F1=2\times \frac{\mathrm{Precision}\times \mathrm{Recall}}{\mathrm{Precision}+\mathrm{Recall}}\;\;, \end{array}$$

where TP denotes True Positives, FP denotes False Positives, TN denotes True Negatives, and FN denotes False Negatives.

Higher values for these metrics indicate better detection performance of the model. The F1-Score is the harmonic mean of Precision and Recall, providing a more balanced performance evaluation. Therefore, the F1-Score is generally considered a more comprehensive evaluation metric, especially suitable for class-imbalanced datasets. All results are presented as percentages (%), with the best results in bold.

#### 4.1.3. Experimental Details

The experimental parameters are summarized as follows. Subsequences were obtained using non-overlapping sliding windows. The sliding window size was fixed at 100 for all datasets, the number of attention heads was 8, the number of residual blocks in the temporal stream STAD-Net was 4, the number of layers in the frequency stream Transformer encoder was 3, the hidden layer dimension of the STAD-Net was 128, the number of masked and unmasked windows in the grating masking strategy was 5 each (the frequency-domain mask ratio $r^{\left(F\right)}\%$ is adjusted according to the anomaly ratio of the corresponding dataset), the number of denoising steps *T* for the diffusion model was set to 50, the trade-off hyperparameter λ in the loss function was set to 1.0, used to balance the temporal imputation loss and the temporal-frequency contrastive loss. The alternating minimization-maximization rounds in adversarial training are set to 100, with an early stopping mechanism implemented. All experiments were implemented in PyTorch version 2.4.0, optimized using the Adam optimizer, with a batch size of 64, an initial learning rate of $10^{-4}$, and trained on a single NVIDIA GeForce RTX 4080 GPU.

### 4.2. Experimental Results (RQ1 & RQ2)

For a comprehensive evaluation, we compared with 10 mainstream baseline methods, covering different technical routes: linear transformation-based methods (OCSVM [56], PCA [57]); density estimation-based methods (HBOS [58]); isolation forest-based methods (IForest [59]); and deep learning-based methods: including Deep Autoencoding Gaussian Mixture Model (DAGMM [60]), temporal networks (LSTM [61]), generative models (BeatGAN [62], OmniAnomaly [1]); the latest Transformer architecture (D3R [63]) and dual contrastive learning-based (DCdetector [39]).

To ensure a fair comparison, all baselines used the same evaluation metrics and threshold determination protocol.

Table 2 presents the detailed performance of all aforementioned methods on the five datasets. First, our model (TFCID) achieves the highest F1 scores across all five real-world multivariate datasets (PSM, SMAP, SWaT, SMD, MSL), with F1 scores exceeding 90% on all datasets and reaching an exceptional 99.64% on the SMD dataset. Notably, on the PSM dataset, our model’s F1 score (98.22%) leads the best baseline method (DCdetector, 96.97%) by 1.25 percentage points, demonstrating a significant advantage. Second, compared to traditional unsupervised methods (e.g., OC-SVM, IFOREST) and deep learning approaches (e.g., LSTM, DAGMM), our model can more effectively learn the complex temporal dependencies inherent in multivariate time series data. Furthermore, compared to reconstruction-based methods such as OmniAnomaly and BeatGAN, the advanced architecture employed in our model significantly enhances the accuracy and robustness of anomaly detection. Compared to the latest methods like DCdetector, our model achieves a better balance between precision and recall while maintaining high recall.

**Figure 5:**
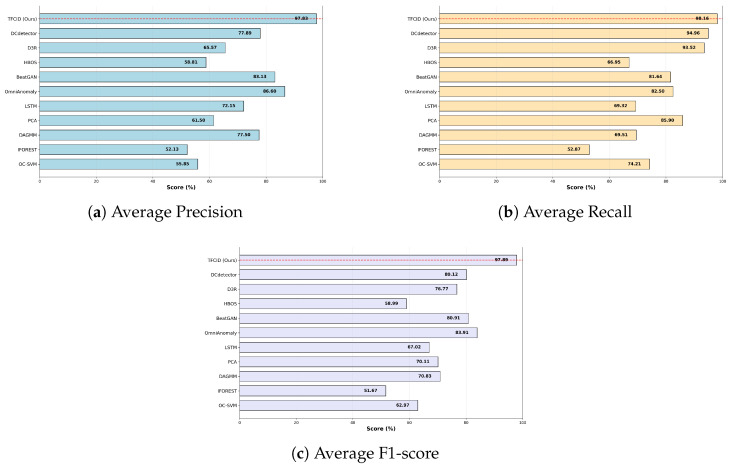
Average performance comparison of each model on the five datasets (PSM, SMAP, SWaT, SMD, MSL).

Figure 5 shows the average performance of all evaluated methods on the five public datasets (PSM, SMAP, SWaT, SMD, MSL). Our model (TFCID) achieves an average F1 score of 97.89%, significantly outperforming other methods. This score exceeds that of the second-best model (OmniAnomaly, F1 = 83.91%) by 13.98 percentage points, demonstrating a substantial advantage. Across all metrics, our model ranks first in both average precision (97.83%) and average recall (98.16%), achieving comprehensive leadership in both indicators. In contrast, other models exhibit varying degrees of performance imbalance: for example, D3R has a relatively high average recall (93.52%) but a low average precision (65.57%); while DCdetector performs well in recall (94.96%), its F1 score is only 80.12%. In summary, our model not only significantly surpasses all baseline methods in F1 score but also achieves the best balance between precision and recall, demonstrating outstanding comprehensive performance.

### 4.3. Anomaly Detection Visualization (RQ3)

**Figure 6:**
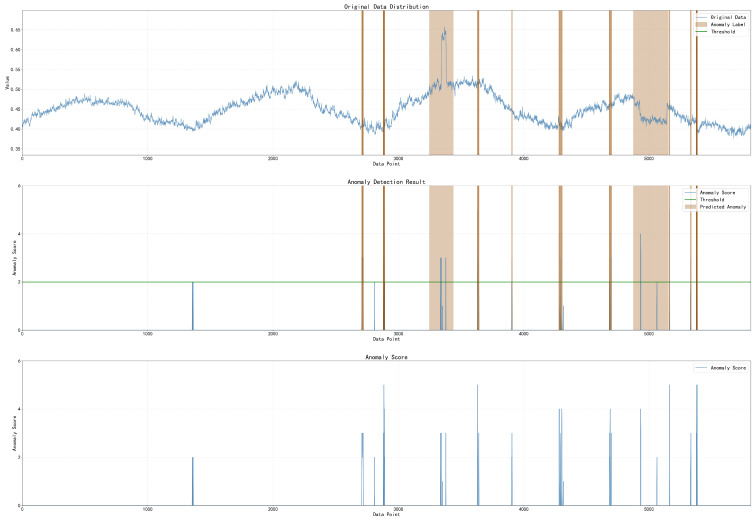
Anomaly detection results of TFCID on a 6000-length sequence from the PSM dataset. The detected anomaly scores (bottom) closely align with the ground-truth anomaly regions (light brown highlights in top), demonstrating accurate identification of both seasonal and global anomalies with low false positives.

To validate the capability of TFCID in generating accurate detection results, we conducted a case study using the PSM dataset. As illustrated in Figure 6, the anomaly scores produced by TFCID remain consistently distinguishable. They stay at low levels except at time points where anomalies are present. More importantly, TFCID successfully identifies both seasonal and global observation anomalies in the multivariate time series dataset PSM, which benefits from the superior sensitivity of the frequency domain to pattern anomalies. Furthermore, from an overall perspective, the effectiveness of TFCID in anomaly detection is evident from the correspondence between the original anomalies and the detected anomalies in Figure 6. TFCID accurately identifies anomalous data points or segments while maintaining low detection scores in normal regions.

### 4.4. Ablation Study (RQ4)

To systematically evaluate the contribution of each component in TFCID, we conducted ablation experiments by removing or replacing key modules. The results in Table 3 reveal a clear hierarchy of importance:

1. The imputation task (w/o imputation) causes the most severe performance degradation (19.81% drop), confirming that bidirectional context modeling is fundamental to the framework.
2. The masking strategy (w/o masking) leads to a 17.78% drop, indicating that forcing the model to reconstruct from partial observations is essential for learning anomaly-sensitive representations.
3. Adversarial training (w/o adversarial training) results in a 10.98% drop, validating its role in mitigating distribution shift.
4. The frequency domain module (w/o frequency domain) causes a 10.65% drop, demonstrating the importance of capturing periodic patterns.
5. The unconditional diffusion design (w/o unconditional diffusion, i.e., replaced with conditional diffusion) yields a 4.73% drop, confirming its advantage in avoiding anomaly contamination.

This ablation hierarchy demonstrates that each module addresses a distinct challenge: imputation for uncertainty reduction, masking for representation learning, frequency analysis for periodic patterns, adversarial training for distribution robustness, and unconditional diffusion for anomaly bias prevention.

#### 4.4.1. Ablation Analysis of Frequency Domain Module

When the frequency domain processing module (w/o frequency domain) is removed, the average F1-score of the model drops from 97.89% to 87.24%, a decrease of 10.65 percentage points. This module aims to extract periodic, trend-related, and other frequency-domain features from time series, and its absence directly impacts the model’s ability to identify structural anomalies. On the SMD dataset, the performance degradation is most significant (13.52 percentage points), which aligns with the dataset’s rich periodic pattern characteristics. This validates the critical role of frequency domain processing in capturing periodic and trend-related anomalies in time series.

#### 4.4.2. Ablation Analysis of Masking Module

When the masking strategy (w/o masking) is removed, the model performance declines by 17.78 percentage points (97.89%→80.11%). The masking operation randomly conceals parts of the input data, forcing the model to learn how to reconstruct the masked content based on contextual information, thereby enhancing sensitivity to anomalous patterns. Without this mechanism, the model tends to degrade into simple data reconstruction, making it difficult to effectively distinguish between normal and anomalous patterns.

#### 4.4.3. Ablation Analysis of Unconditional vs. Conditional Diffusion

To validate the advantage of our unconditional diffusion design over the conditional alternative, we conducted a comparative experiment where the unconditional diffusion module in TFCID was replaced with a conditional diffusion design (similar to CSDI [13]), denoted as “w/o unconditional diffusion”. As shown in Table 3, this conditional variant achieves an average F1-score of 93.16%, which is 4.73 percentage points lower than our unconditional design (97.89%). This result indicates that unconditional diffusion plays a key role in constructing a clear boundary between normal and anomalous patterns. The conditional model tends to directly use input samples (which may contain potential anomalies) as guiding signals, making it prone to mistakenly treating anomalous patterns in the training data as normal features. This leads to overfitting and weakens the model’s generalization ability on unseen data. Unconditional diffusion, by contrast, does not rely on the original input as a condition but reconstructs the data distribution step-by-step through noise, indirectly leveraging contextual information during the denoising process. It thereby demonstrates stronger robustness and discriminative power for anomalous samples. The performance gap is particularly pronounced on datasets with higher anomaly rates, where conditional models are more susceptible to anomaly contamination during training. This empirical result corroborates our theoretical analysis in Section 3.3.2.

#### 4.4.4. Ablation Analysis of Imputation Module

When the imputation task is replaced with a direct prediction task (w/o imputation), performance decreases by 19.81 percentage points (97.89%→78.08%). The imputation task can leverage bidirectional contextual information around the masked positions, resulting in lower uncertainty. In contrast, the prediction task relies solely on historical information, with future information entirely unknown, making modeling more challenging and leading to inferior performance in anomaly detection scenarios.

#### 4.4.5. Ablation Analysis of Adversarial Training Mechanism

When the adversarial training module (w/o adversarial training) is removed, performance declines by 10.98 percentage points (97.89%→86.91%). Adversarial training enhances the distinction between temporal and frequency domain representations through gradient reversal layers and dual discriminators, improving the model’s adaptability to distributional shifts.

Adversarial loss function:

(18) $$L_{adv}=E_{z_{time}∼P_{enc}\left(z|x\right)}\left[\log D\left(z_{time}\right)\right]+E_{z_{freq}∼P_{enc}\left(z|x\right)}\left[\log\left(1-D\left(z_{freq}\right)\right)\right]\;$$

Adversarial training significantly enhances the discriminability between temporal and frequency domain representations through gradient reversal layers and dual discriminators:

(19) $$\frac{\partial L_{adv}}{\partial \theta_{f}}=-\lambda \frac{\partial L_{disc}}{\partial \theta_{f}}\;$$

During the adversarial training phase, the model optimizes the domain discriminator *D* by minimizing the adversarial loss $L_{adv}$, while simultaneously optimizing the encoder parameters $\theta_{f}$ using a gradient reversal layer (GRL). Here, $z_{time}$ and $z_{freq}$ represent the temporal and frequency domain latent representations extracted by the encoder from the input data *D*, with their distribution defined by $P_{enc}\left(z|x\right)$. The expectation E denotes the mathematical expectation over all extracted features. The discriminator D(·) aims to accurately distinguish the source of features by maximizing the predicted probability $\log D(z_{time})$ for temporal features and minimizing the misclassification of frequency domain features. During backpropagation, the gradient reversal layer applies a scaling factor λ to invert the gradient of the discriminator loss, compelling the encoder to generate highly discriminative temporal and frequency domain features.

This component effectively enhances the model’s robustness to distribution shifts, particularly when facing unseen anomaly patterns, contributing to a performance improvement of 14.13 percentage points on the SMD dataset.

#### 4.4.6. Analysis of Component Synergy

The experimental results reveal significant synergistic effects among the modules: the dual-stream temporal and frequency domain architecture captures anomalies from different perspectives; the masking strategy provides a more challenging learning objective for the diffusion model; and adversarial training, combined with contrastive learning, jointly enhances the model’s discriminative power and robustness.

The full model achieves optimal performance across all datasets (PSM: 98.22%, SMAP: 97.86%, SWaT: 97.88%, SMD: 99.64%, MSL: 95.85%), demonstrating the rationality and necessity of each component design. Particularly on the SMD dataset, which contains complex periodic patterns, the model achieves a performance of 99.64%, highlighting its effectiveness in handling periodic anomalies. On the PSM and MSL datasets, the contributions of each module are relatively balanced, showcasing the model’s generalizability. The ablation experiments not only validate the effectiveness of each independent module but also reveal their synergistic mechanisms, providing valuable insights for the design of time series anomaly detection models.

### 4.5. Hyperparameter Analysis (RQ5)

**Figure 7:**
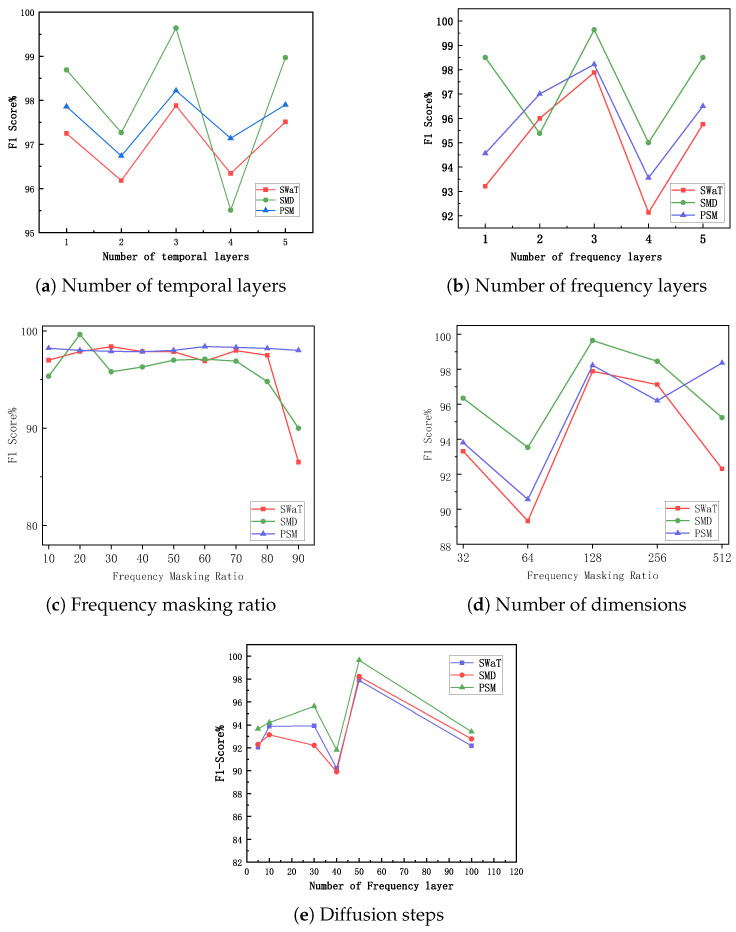
Parameter sensitivity analysis.

We investigated the impact of key hyperparameters, as shown in Figure 7.

- **Number of Temporal Transformer Layers (Figure 7a) and Number of Frequency Transformer (Figure 7b)**: Both temporal and frequency domains show similar trends: the model achieves the best performance balance when the number of layers is set to 3. Deeper networks may lead to overfitting.
- **Frequency Masking Ratio (Figure 7c):** The model is more sensitive to the frequency masking ratio because a single frequency contains richer information than a single time point. The optimal ratio is typically between 10–40%.
- **Embedding Dimension (Figure 7d):** Overall, the model performance remains stable with respect to the embedding dimension. A dimension of 128 provides a good balance between performance and efficiency.
- **Number of Diffusion Denoising Steps (Figure 7e):** The number of diffusion denoising steps has a significant impact on model performance. As the number of steps increases from 5 to 50, the F1 score continuously improves, indicating that more denoising steps help generate higher-quality imputation results. However, when the number of steps exceeds 50, the performance gain saturates, while the computational cost increases significantly.

Based on the parameter sensitivity study shown in Figure 7, we conducted a systematic analysis of the key hyperparameters of the TFCID model. First, the number of layers for both the Temporal and Frequency Transformers (Figure 7a,b) shows similar influence trends: the model reaches the performance peak on most datasets when the number of layers is set to 3. Too few layers (e.g., 1 layer) may limit the model’s ability to capture complex temporal or frequency domain dependencies, while too many layers (e.g., 5 layers) may lead to overfitting and significantly increase the computational burden. Thus, 3 layers prove to be a robust choice that balances expressive power and efficiency. Second, the Frequency Masking Ratio (Figure 7c) is a relatively sensitive hyperparameter, with an optimal range typically between 10% and 40%. A ratio that is too low may cause the model to learn too many frequency components containing noise, while a ratio that is too high will lose key periodic pattern information, harming the learning effect of the frequency stream. In contrast, the Embedding Dimension (Figure 7d) has a relatively mild impact on model performance; the F1 score fluctuates slightly within a wide range from 32 to 512, indicating that the model has good robustness to this parameter; for a comprehensive trade-off between computational memory and representational capacity, we finally chose 128 as the default dimension. Finally, the number of denoising steps for the diffusion model (Figure 7e) is crucial for the imputation quality: as the number of steps increases from 5 to 50, the F1 score shows a significant and continuous improvement, confirming that a finer gradual denoising process helps generate more accurate and natural sequence imputation results; however, when the number of steps exceeds 50, the performance gain tends to saturate, while the inference time increases linearly, so we set the number of diffusion steps to 50 to achieve the optimal balance between performance and efficiency. Overall, these analyses provide an empirical basis for the selection of model hyperparameters, ensuring the stable and efficient performance of TFCID across different datasets.

## 5. Conclusions

This paper proposed TFCID, an innovative Temporal-Frequency Contrastive Imputation Diffusion framework for multivariate time series anomaly detection. The model effectively addresses the challenges of anomaly bias and distribution shift faced by traditional methods by integrating the precise imputation capability of unconditional diffusion models, the complementary information of temporal-frequency dual-stream views, and the distribution-invariant properties of adversarial contrastive learning. Experimental results on multiple public datasets demonstrate that TFCID significantly outperforms existing state-of-the-art methods in terms of detection accuracy and robustness. Future work will focus on the lightweight design of the model to further improve efficiency and explore its application potential in broader temporal analysis tasks (e.g., forecasting and classification).

Limitations and Practical Deployment Challenges: Despite the strong performance of TFCID, several limitations warrant discussion. First, computational overhead: The diffusion model requires multiple denoising steps (T = 50 in our experiments), which increases inference latency compared to single-pass methods. On an NVIDIA RTX 4080 GPU, processing a batch of 64 samples with sequence length 100 takes approximately 0.5 s per diffusion step, resulting in 25 s per batch. This may be prohibitive for real-time applications with strict latency requirements (e.g., sub-second industrial control loops). Second, hyperparameter sensitivity: While TFCID achieves robust performance across datasets, optimal hyperparameters (e.g., frequency masking ratio, number of diffusion steps) vary with data characteristics, requiring dataset-specific tuning. Third, anomaly interpretation: TFCID provides anomaly scores but does not inherently offer interpretability regarding which variables or time steps contribute most to a detected anomaly, which is critical for root cause analysis in industrial settings. Future work will focus on model compression (e.g., diffusion step distillation), adaptive hyperparameter selection, and integrating explainability mechanisms to address these limitations.

## Figures and Tables

**Table 1:** Dataset Information.

Dataset	Dimensions	Training Set	Validation Set	Test Set	Anomaly Rate (%)
SMD	38	566,724	141,681	708,420	4.2
PSM	25	105,984	26,497	87,841	27.75
SWaT	51	396,000	99,000	449,919	11.98
MSL	55	46,653	11,664	73,729	10.5
SMAP	25	108,146	27,037	427,617	13.13

**Table 2:** Overall Results on Real-World Multivariate Datasets (PSM, SMAP, SWaT, SMD, MSL). Precision (P), Recall (R), and F1 scores are used as evaluation metrics. All results are in %. For these three metrics, higher values indicate better performance, and the best ones are in bold.

Dataset	PSM				SMAP				SWaT				SMD				MSL		
Metric	P	R	F1		P	R	F1		P	R	F1		P	R	F1		P	R	F1
OC-SVM	62.75	80.89	70.67		53.85	59.07	56.34		45.39	49.22	47.23		66.98	82.03	73.75		50.26	**99.86**	66.87
IFOREST	76.09	92.45	83.48		52.39	52.39	55.53		49.29	44.95	47.02		20.30	21.30	17.99		60.59	53.28	53.34
DAGMM	93.49	70.03	80.08		86.45	56.73	68.51		89.92	57.84	70.40		63.57	70.83	67.00		54.07	92.11	68.14
PCA	77.44	63.68	69.89		50.62	98.48	66.87		62.32	82.96	71.18		64.42	86.06	74.01		52.69	98.33	68.61
LSTM	73.62	89.92	80.96		92.20	67.75	78.10		76.00	89.50	82.20		60.12	84.77	70.35		58.82	14.68	23.49
OmniAnomaly	88.39	74.46	80.83		92.49	81.99	86.92		81.42	84.30	82.83		87.51	90.52	87.75		83.21	81.25	82.21
BeatGAN	90.30	93.84	92.04		92.38	55.85	69.61		64.01	87.46	73.92		90.13	88.94	87.97		77.82	85.12	81.02
HBOS	78.45	29.82	43.21		41.54	66.17	51.04		54.49	91.35	68.26		60.34	64.11	62.17		59.25	83.32	69.25
D3R	73.32	88.71	80.29		61.76	92.55	74.09		61.04	97.57	74.41		64.87	97.93	78.04		66.85	90.83	77.02
DCdetector	96.62	97.32	96.97		94.44	97.80	96.09		92.53	88.59	90.52		50.93	95.57	66.45		55.94	95.53	70.56
**TFCID (Ours)**	**99.24**	**98.59**	**98.22**		**99.71**	**99.12**	**97.86**		**99.69**	**99.06**	**97.88**		**99.28**	**99.98**	**99.64**		**91.25**	94.06	**95.85**

**Table 3:** Performance Comparison of Ablation Studies. All results are in %. For the F1-score metric, higher values indicate better performance, and the best values are highlighted in bold.

Method	F1-Score					
	PSM	SMAP	SWaT	SMD	MSL	Avg.
w/o adversarial training	88.22	87.86	87.88	85.51	85.09	86.91
w/o imputation	79.86	78.66	78.68	76.78	76.45	78.08
w/o unconditional diffusion	93.35	94.54	93.56	93.15	91.22	93.16
w/o masking	81.23	80.93	80.95	78.99	78.43	80.11
w/o frequency domain	88.54	88.21	88.23	86.12	85.12	87.24
TFCID (ours)	**98.22**	**97.86**	**97.88**	**99.64**	**95.85**	**97.89**

## Data Availability

The original contributions presented in this study are included in the article. Further inquiries can be directed to the corresponding author.
